# Macrophage–Myocyte Cross Talk Induces M1 Polarization and Inflammatory Cytokine Production During Cancer Cachexia

**DOI:** 10.1155/mi/6461737

**Published:** 2026-07-14

**Authors:** Se-Eun Bae, Miyong Yun, Min Ju Kang, Sang Woo Woo, Do Won Kim, Eun-Ok Kim, Hyeon Soo Kim

**Affiliations:** ^1^ Department of Anatomy, Korea University College of Medicine, Seoul, Republic of Korea, korea.ac.kr; ^2^ Department of Bioindustry and Bioresource Engineering, Sejong University, Seoul, Republic of Korea, sejong.ac.kr; ^3^ Medical Science Research Center, Korea University College of Medicine, Seoul, Republic of Korea, korea.ac.kr

## Abstract

Cancer cachexia (CC) significantly reduces survival in patients with cancer, and intramuscular inflammation plays an important role in disease progression. Macrophages, as key mediators of the innate immune response, contribute to muscle inflammation by secreting pro‐inflammatory cytokines, including interleukin (IL)‐1β, IL‐6, and tumor necrosis factor‐alpha (TNF‐α), in response to various stimuli. Despite their importance, the mechanisms of macrophage polarization within the cachectic skeletal muscle and the subsequent impacts on muscle inflammation remain poorly understood. In this study, we used a murine model of CC to explore the interaction between macrophages and myocytes during muscle inflammation. Our results demonstrate that cachectic myocytes recruit M1‐polarized macrophages that amplify local inflammation via the secretion of IL‐1β, IL‐6, and TNF‐α. These findings underscore the pivotal role of macrophage‐derived cytokines in the pathogenesis of cancer‐induced muscle inflammation and suggest that targeting macrophage‐driven inflammatory pathways may be an effective therapeutic strategy for mitigating muscle wasting in CC.

## 1. Introduction

Cancer cachexia (CC) is a complex metabolic syndrome characterized by progressive weight loss, anorexia, and skeletal muscle and adipose tissue wasting. It is a common complication of advanced cancer and is strongly associated with poor prognosis, accounting for up to 20% of cancer‐related deaths [1, 2]. The development of CC is driven by multiple factors, including systemic inflammation and altered metabolism, all of which contribute to subsequent muscle wasting [2, 3]. Persistent anorexia and weight loss affect over 50% of patients with advanced cancer, highlighting the urgent need for effective therapeutic interventions [4]. Historically, CC has been attributed to decreased protein synthesis and increased muscle catabolism [5]. Early treatments aimed at muscle preservation yielded limited success, leading to a shift toward more comprehensive approaches that address inflammation, metabolism, and nutrition [5–8]. Despite these efforts, progress remains slow due to only a partial understanding of the underlying mechanisms.

Pro‐inflammatory cytokines, including tumor necrosis factor (TNF)‐α, interleukin (IL)‐1β, IL‐6, and IL‐8, are critical mediators of muscle wasting in CC [3, 9–12]. These cytokines, produced by tumors and immune cells, disrupt metabolic homeostasis and activate signaling pathways that promote muscle degradation. Consequently, immunological interventions targeting inflammatory cytokines have gained attention as potential therapeutic options for CC [13–17]. However, the exact mechanisms underlying the inflammatory response in CC and the most effective strategies to target this pathway remain unclear. A more detailed understanding of the role of the immune system in muscle wasting, particularly the impact of soluble inflammatory mediators, is essential for developing novel therapies.

Although immune cells comprise only 2%–6% of skeletal muscle cells, they are essential in maintaining muscle homeostasis. Among these immune cells, macrophages are key regulators of inflammation [18]. Macrophages exhibit remarkable phenotypic plasticity, adopting diverse activation states in response to complex microenvironmental cues [19, 20]. Although the classical M1/M2 paradigm has historically provided a useful framework for categorizing pro‐ and anti‐inflammatory macrophage phenotypes in vitro [21–23], macrophage activation in vivo is far more nuanced, occurring along a continuous functional spectrum rather than in binary states [24, 25]. In cancer, macrophages have been implicated in the promotion of myotube atrophy, and their depletion has been shown to attenuate cachexia and systemic inflammation [26].

However, the mechanisms of macrophage polarization within cachectic skeletal muscles and how these interactions drive muscle inflammation remain poorly understood. In this study, we used a well‐established colon‐26 (C26) adenocarcinoma–induced cachexia model [27] to investigate the effects of macrophage polarization on cytokine production by cachectic myocytes. We also examined how cachectic myocytes influence macrophage polarization to elucidate the bidirectional cross talk between macrophages and muscle cells during the progression of CC. Our findings provide novel insights into the inflammatory axis governing skeletal muscle wasting in patients with CC.

## 2. Materials and Methods

### 2.1. Experimental Animals and CC Model Design

Approval for animal experiments was obtained from the Korea University Institutional Animal Care and Use Committee (KOREA‐2023‐0015). All procedures adhered to the relevant guidelines and regulations, following the recommendations outlined in the 8th Edition of The Guide for the Care and Use of Laboratory Animals by the National Research Council Committee. Male BALB/c mice, aged 8 weeks, were purchased from Koatech (Pyeongtaek, Republic of Korea) and maintained under standardized conditions, including a temperature range of 21–23°C, a relative humidity of 50%–60%, and a 12‐h/12‐h light/dark cycle. Mice were provided with ad libitum access to a standard laboratory chow diet and water throughout the study. Following acclimation, the animals were randomly assigned to two experimental groups: the control and C26 tumor‐bearing groups (*n* = 15 per group). To ensure baseline uniformity, group allocation was performed using a weight‐stratified randomization method, such that the initial mean body weight and variance were statistically indistinguishable between groups.

At 9 weeks of age, each mouse was subcutaneously injected with either 2.0 × 10^6^ C26 tumor cells suspended in 100 µL of sterilized phosphate‐buffered saline (PBS) or 100 µL of sterilized PBS alone (control group). The body weights of the mice were monitored weekly following tumor inoculation. Six weeks after inoculation, the mice were euthanized, and the skeletal muscles, specifically the soleus (Sol), extensor digitorum longus (EDL), gastrocnemius (GC), tibialis anterior (TA), and quadriceps (QC), were carefully excised. The collected muscle tissues were immediately rinsed with ice‐cold PBS to remove residual blood, carefully weighed, and subsequently processed for downstream applications. For flow cytometric analysis, the QC muscle underwent enzymatic and mechanical dissociation rather than standard homogenization to preserve cellular integrity.

**Figure 1:**
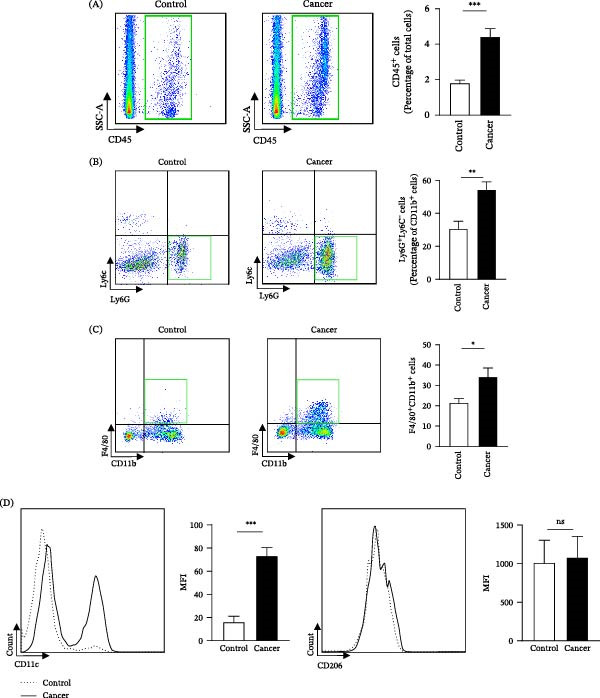
Myeloid cell profiling in skeletal muscle from cancer cachexia (CC) mice. BALB/c mice were randomly assigned to control and C26 tumor‐bearing groups (*n* = 15 per group), C26 tumor cells were subcutaneously injected to induce CC, and the quadriceps (QC) muscles were harvested on day 42 postinoculation. Flow cytometry was performed to quantify (A) CD45^+^ leukocytes, (B) neutrophils (CD45^+^CD11b^+^Ly6G^+^Ly6C^lo^), and (C) macrophages (CD45^+^CD11b^+^F4/80^+^) in the skeletal muscle tissue. Representative gating boundaries used for quantification are indicated by green boxes in each plot. (D) The expression of M1 (CD11c^+^) and M2 (CD206^+^) markers was analyzed within the macrophage population gated in Panel (C). Solid and dashed lines in the histograms indicate mean fluorescence intensity (MFI) for cancer and control groups, respectively. Bars represent the mean values, and the error bars indicate the standard error of the mean (SEM). Statistical significance was determined using unpaired Student’s *t*‐test or Welch’s *t*‐test, depending on the equality of variances. Data are indicated as follows:  ^∗^ *p* < 0.05;  ^∗∗^ *p* < 0.01;  ^∗∗∗^ *p* < 0.001; ns = not significant, *p*  > 0.05.

Among the excised muscles, the QC muscle was selected for flow cytometric analysis (Figure 1) because of its sufficient tissue volume and well‐documented high susceptibility to cachexia‐induced atrophy in the C26 model. Data regarding the mass of all excised muscles (Sol, EDL, GC, TA, and QC) are provided in Figure S1C to demonstrate the extent of systemic muscle wasting.

### 2.2. In Vivo Muscle Grip Strength Measurements

To evaluate the grip strength of the forelimbs and hind limbs (all four paws of the mice), a calibrated grip strength tester (BIOSEB, BIO‐GS3) was used [27]. For each mouse, grip strength was measured thrice, and the data were expressed as the mean value.

### 2.3. Cell Culture

C26 colon carcinoma cells (RRID: CVCL_0240) were maintained in RPMI 1640 medium supplemented with 10% fetal bovine serum (FBS; Gibco, Grand Island, NY, USA) and 1% penicillin and streptomycin (WELGENE, Kyeongsan, Republic of Korea) at 37°C in a humidified incubator with 5% CO_2_. Prior to injection into mice (day 0), the cells were collected and resuspended at a concentration of 2 × 10^6^ cells/mL in sterilized PBS. C2C12 murine skeletal muscle precursor cells (myoblasts, RRID: CVCL_0188) were maintained in a growth medium (GM) consisting of Dulbecco’s modified Eagle’s medium (DMEM) supplemented with 10% FBS (Gibco) and 1% penicillin and streptomycin (WELGENE, Kyeongsan, Republic of Korea) at 37°C in a humidified incubator with 5% CO_2_.

To induce differentiation, the medium was replaced with differentiation medium (DM:DMEM supplemented with 2% horse serum) for 5 days [28]. To prepare the C26 cancer cell‐conditioned media (C26‐CM), C26 cells were cultured in serum‐free DMEM for 72 h. The supernatant was then harvested and filtered through a 0.2 μm‐pore‐sized filter (Merck Millipore, Burlington, MA, USA) to remove cellular debris.

To assess the impact of myotube‐derived factors on macrophage polarization, C2C12 myotubes were treated with either DM (as a baseline) or 20% C26‐CM for 24 h to generate “C2C12 DM sup” and “C2C12 CM sup,” respectively. For BMDM stimulation experiments, all groups (Control, C2C12 DM sup, and C2C12 CM sup) were maintained in serum‐free and M‐CSF‐free DMEM during the 24‐h treatment period. The “control” group was incubated with serum‐free, M‐CSF–free DMEM alone.

This experimental design ensured that observed effects on BMDM polarization were driven solely by factors released from the myotubes, eliminating any potential confounding effects from exogenous growth factors (M‐CSF) or serum components (FBS). All in vitro experiments were performed using at least three independent biological replicates (*n* = 3 per group).

### 2.4. Muscle Dissociation

The muscle tissue was dissected, minced, and enzymatically disaggregated in 4 mL of PBS containing 1.5 U/mL dispase II and 1.4 U/mL collagenase D (Roche, Basel, Switzerland) at 37°C. The mixture was gently triturated using a 10 mL pipette at 5 min intervals for 20 min. Following dissociation, the cell suspension was filtered through a 70 µm strainer (BD Falcon, San Jose, CA, USA) to remove debris. The filtrate was then collected via centrifugation at 200 × *g* for 5 min.

### 2.5. Isolation and Stimulation of Bone Marrow‐Derived Macrophages (BMDMs)

Primary bone marrow was isolated from 6–8‐week‐old BALB/c mice. The hind legs were excised, and the muscles were carefully removed. The leg bones were clamped with forceps, and the joints at both ends were excised. A syringe filled with DMEM was used to flush the marrow cells from the bone. The collected cells were centrifuged at 400 × *g* for 5 min, and the resulting pellet was collected. To lyse red blood cells, 1 mL of ammonium–chloride–potassium (ACK) lysis buffer was added and incubated for 2 min, followed by 3 mL of DMEM. The cell suspension was centrifuged at 400 × *g* for 5 min.

Bone marrow cells were then cultured in DMEM supplemented with 10% FBS, 50 ng/mL recombinant macrophage colony‐stimulating factor (M‐CSF; Prospec Bio., Ness‐Ziona, Israel), and 1% penicillin–streptomycin solution. The medium and non‐adherent cells were discarded the following day, whereas adherent cells were maintained in M‐CSF‐supplemented medium until confluence was reached (5–7 days).

For macrophage polarization, BMDMs were stimulated for 24 h with lipopolysaccharide (LPS; 100 ng/mL; Sigma–Aldrich, St. Louis, MO, USA) and interferon‐γ (IFN‐γ; 20 ng/mL; Prospec Bio.) for classical activation or with IL‐4 (20 ng/mL; Prospec Bio.) and IL‐13 (20 ng/mL; Prospec Bio.) for alternative activation. Following the polarization period, the stimulation media were completely removed. To eliminate any residual polarization stimuli, the cells were washed three times with PBS and incubated in fresh culture media for an additional 2 h. The supernatants were then collected and used for the in vitro CC model. This washing and medium replacement protocol ensured that the observed effects were exclusively attributed to the factors secreted by the polarized macrophages without interference from the initial stimulants. All in vitro experiments were performed using at least three independent biological replicates (*n* = 3 per group).

### 2.6. Flow Cytometry

Collected cells were treated with BD Fc Block Receptor Binding Inhibitor (BD Biosciences, San Jose, CA, USA) for 5 min at room temperature. Subsequently, the cells were stained with fluorochrome‐conjugated antibodies for 20 min at 4°C in the dark. After staining, the labeled cells were washed and centrifuged at 400 × *g* for 5 min, followed by resuspension in 300 µL of fluorescence‐activated cell sorting (FACS) buffer containing PBS and 5% FBS. Flow cytometry was performed using a Cytek Aurora spectral flow cytometer (Cytek Biosciences, Fremont, CA, USA), FACS Canto II (BD Biosciences, San Jose, CA, USA), and Aria III (BD Biosciences, San Jose, CA, USA). Flow cytometry was performed using three different instruments. To optimize the detection of fluorochromes, instruments with laser configurations that best matched the antibody panels were selected. Despite the use of different instruments, identical antibody clones, staining protocols, and gating strategies were strictly applied to ensure data comparability across all experimental groups. Data were analyzed using FlowJo software v10 (Treestar, Ashland, OR, USA).

The antibodies used to detect immune cell subsets in muscle tissues are listed in Table 1. Leukocytes were identified as CD45^+^ cells, whereas myeloid cells were characterized as CD45^+^CD11b^+^. Ly6G^+^ cells were defined as neutrophils, and Ly6G^−^ myeloid cells were further classified as F4/80^+^ macrophages. To analyze macrophage polarization, the following antibodies were used: anti‐CD11c‐Alexa 700 and anti‐CD206‐APC.

**Table 1:** List of antibodies used in the study.

Antibody	Fluorescence	Ca #	Clone	Dilution ratio	Vendor
Viability	BV510	62910‐00	62910‐00	1 µL/sample	BioGerm
CD3	PE‐cy7	25‐0031	145‐2C11	1:40	eBioscience
CD19	BV421	115537	6D5	1:100	Biolegend
CD45	APC cy7	103116	30‐F11	1:50	Biolegend
Ly6C	FITC	128006	HK1.4	1:50	eBioscience
Ly6G	BV711	127627	1A8	1:50	Biolegend
F4/80	BV421	123131	BM8	1:50	Biolegend
CD11b	PerCPcy5.5	101228	M1/7	1:50	Biolegend
MHC II	PE cy7	116420	AF6‐120.1	1:80	Biolegend
CD11c	Alexa 700	117320	N418	1:50	Biolegend
CD206	APC	17‐2061‐82	MR6F3	1:50	eBioscience

### 2.7. RNA Isolation and Quantitative Reverse‐Transcription Polymerase Chain Reaction (qRT‐PCR)

Total RNA was extracted from muscle tissue, C2C12 myotubes, and primary macrophages using the Qiazol reagent (Qiagen, Valencia, CA, USA), in accordance with the manufacturer’s protocol. RNA concentrations and purity were assessed using a NanoDrop spectrophotometer (Thermo Fisher Scientific, Waltham, MA, USA). The A260/280 ratio for all samples consistently fell within the range of 1.8–2.0, ensuring high purity for downstream applications. cDNA synthesis was performed using a reverse transcription system (Promega, Madison, WI, USA). qRT‐PCR was performed using a QuantStudio 3 Real‐Time PCR system (Thermo Fisher Scientific) with the SYBR Green kit (TOPreal qPCR 2X PreMIX, Enzynomics, Daejeon, Republic of Korea). The primer sequences used for the analysis are listed in Table 2. Real‐time PCR amplification was performed with an initial denaturation at 95°C for 10 min, followed by 40 cycles of 95°C for 15 s and 60°C for 1 min. To ensure specificity, a melt curve analysis was performed after the run. For in vivo samples, representative muscle tissues were randomly selected for analysis from the full cohort (*n* = 15 per group) after confirming successful model induction based on phenotypic consistency and RNA quality.

**Table 2:** List of primers used in the study.

Gene (species)	Sequence 5→3	
*GAPDH* (mouse)	Fw	GTG TTC CTA CCC CCA ATG TG
	Rev	CCT GCT TCA CCA CCT TCT TG
*Myostatin* (mouse)	Fw	GCG ATG AGC ACT CCA CGG AA
	Rev	GCC TCT GGG GTT TGC TTG GT
*MurF1* (mouse)	Fw	GTC CAT GTC TGG AGG TCG TT
	Rev	AGG AGC AAG TAG GCA CCT CA
*Atrogin-1* (*Fbxo32/MAFbx*) (mouse)	Fw	CAA GTC TGT GCT GGT GGG CA
	Rev	GGC AGG TCG GTG ATC GTG AG
*IL-1b* (mouse)	Fw	AGG AGA ACC AAG CAA CGA CA
	Rev	TGG GTG TGC CGT CTT TCA TT
*IL-6* (mouse)	Fw	CCA GTT GCC TTC TTG GGA CT
	Rev	GTG AAG TAG GGA AGG CCG TG
*TGF-b* (mouse)	Fw	TCG CTT TGT ACA ACA GCA CC
	Rev	ACT GCT TCC CGA ATG TCT GA

All experiments were analyzed with at least three independent biological replicates, each performed in technical triplicate. The relative expression levels of target genes were normalized to the housekeeping gene, *GAPDH*. GAPDH expression stability was confirmed across all experimental groups prior to the analysis. Fold change values were calculated using the ^ΔΔ^Ct method.

### 2.8. Statistical Analysis

Data are expressed as the mean ± standard error of the mean (SEM). All data were first tested for normality using the Shapiro–Wilk test. For the comparison of two independent groups, the equality of variances was evaluated using the *F*‐test. If the variances were significantly different (*p* < 0.05), Welch’s *t*‐test was applied; otherwise, a standard Student’s *t*‐test was used. For experiments involving more than two groups, one‐way ANOVA followed by the Dunnett’s post hoc test was performed for multiple comparisons. Statistical significance was set at *p* < 0.05. Statistical analyses were performed using GraphPad Prism 9 (GraphPad software, San Diego, CA, USA), and *p*‐values < 0.05 were considered statistically significant.

## 3. Results

### 3.1. C26 Tumor Inoculation Induces Cachexia‐Associated Muscle Atrophy

To establish a representative CC model, BALB/c mice were subcutaneously inoculated with C26 colon adenocarcinoma cells, as previously described [29, 30]. Tumor‐bearing and control mice were monitored weekly for changes in body weight and grip strength over 6 weeks. At 6 weeks postinoculation, the body weights of tumor‐bearing mice (including the tumor) increased to 124.6% ± 0.67% of baseline, compared to a 111.7% ± 0.52% increase in the control mice. This difference was primarily attributed to tumor growth (Figure S1A). Despite the weight gain, functional assessment revealed a significant decline in muscle strength. Both the 2‐ and 4‐limb grip strength measurements were markedly reduced in tumor‐bearing mice relative to controls (Figure S1B). To assess skeletal muscle atrophy, leg skeletal muscles were dissected postmortem and grouped as QC, TA + EDL, and GC plus soleus (GC + Sol). The weights of all muscle groups were significantly lower in tumor‐bearing mice than those in the controls (Figure S1C, left). The total hindlimb muscle mass was also significantly reduced (Figure S1C, right). At the molecular level, the expression of key muscle atrophy markers‐muscle RING‐finger protein (MuRF), myostatin (MSTN), and atrogin‐1‐was significantly elevated in the skeletal muscle of tumor‐bearing mice, with approximately 2.5‐, 2‐, and 3‐fold increases, respectively, compared to controls (Figure S1D). Together, the reductions in muscle mass and function, along with the upregulation of atrophy‐related genes, confirm the successful establishment of a CC phenotype in this murine model.

### 3.2. Macrophage Infiltration and M1 Polarization Characterize Cachectic Muscle Inflammation

CC is marked by progressive muscle wasting and systemic inflammation [31]. To assess immune cell infiltration in the skeletal muscles of cachectic mice, flow cytometric analysis was performed. The proportion of CD45‐positive leukocytes (excluding dead cells) was significantly elevated in tumor‐bearing mice, reaching 4.4% ± 0.42%, compared to 1.8% ± 0.15% in the controls (Figure 1A). This increase was largely because of an expanded population of cells with relatively high side scatter characteristics, indicative of myeloid lineage cells. Further analysis of myeloid subsets revealed a marked increase in neutrophils, identified as CD45^+^CD11b^+^Ly6G + Ly6C^low (Figure 1B). In tumor‐bearing mice, neutrophils constituted 54.33% ± 4.76% of CD45^+^ cells, nearly twice the proportion observed in controls (30.7% ± 4.53%), consistent with reports of neutrophilia in patients with CC [32]. Macrophages, identified as CD45^+^CD11b^+^F4/80^+^ cells, were also significantly increased in the cancer group, rising from 21.5% ± 1.99% to 34.2% ± 4.36% of CD45^+^ cells (Figure 1C). In contrast, lymphocyte populations—including B cells (CD45^+^CD19^+^) and T cells (CD45^+^CD3^+^)—did not differ significantly between groups, suggesting minimal lymphocyte involvement in CC–associated muscle inflammation (Figure S2). To characterize macrophage polarization, the expression of CD11c and CD206, M1 and M2 markers, respectively, was evaluated [33]. Tumor‐bearing mice exhibited a substantial increase in CD11c^+^ macrophages, along with elevated mean fluorescence intensity (MFI), indicating M1 polarization (Figure 1D). In contrast, CD206 expression showed no significant change, suggesting a shift toward pro‐inflammatory macrophage phenotypes in the cachectic muscle.

### 3.3. Cachectic Myocytes Promote M1 Macrophage Polarization via Soluble Mediators

**Figure 2:**
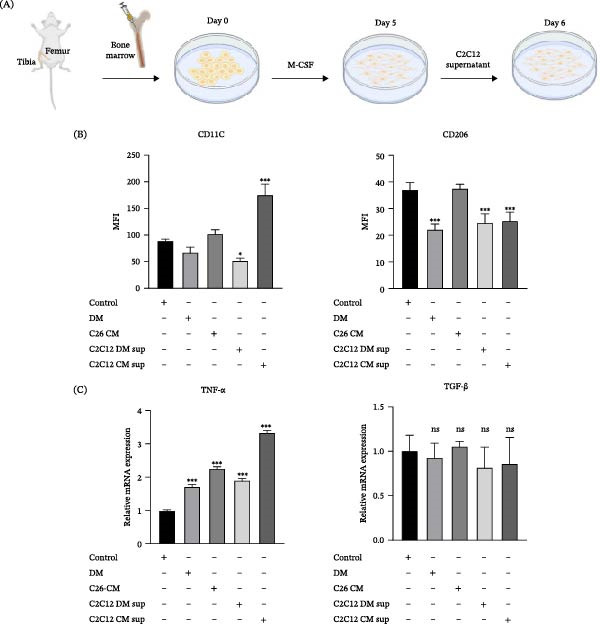
Cachectic myotube‐derived soluble mediators promote M1 macrophage polarization. (A) Schematic diagram of the in vitro experimental design. BMDMs were differentiated with M‐CSF for 5 days and then treated for 24 h with following media: Control (serum‐free/M‐CSF‐free DMEM), DM (C2C12 differentiation medium), C26‐CM (20% cancer cell‐conditioned medium), C2C12 DM sup (supernatant from C2C12 myotubes), and C2C12 CM sup (supernatant from cachectic C2C12 myotubes. (B, C) BMDMs were analyzed for polarization markers via flow cytometry (B) and qRT‐PCR (C). All groups were maintained in serum‐free and M‐CSF‐free conditions during the 24 h treatment. Relative mRNA expression levels were normalized to the mean of the control group using the 2^ΔΔCt^ method, allowing for the inclusion of variance within the control group. Data are expressed as mean ± SEM of three independent experiments (*n* = 3). Statistical significance was determined by one‐way ANOVA followed by the Dunnett’s post hoc test ( ^∗^ *p* < 0.05 and  ^∗∗∗^ *p* < 0.001; ns = not significant).

To determine whether cachectic myocytes contribute to M1 macrophage polarization, an in vitro coculture system was established using BMDMs exposed to conditioned supernatants from C2C12 cells (Figure 2A). To isolate the specific impact of myotube‐derived factors while ensuring rigorous experimental control, we defined three primary treatment groups: control, consisting of serum‐free, M‐CSF‐free DMEM; C2C12 DM sup, the supernatant from differentiated C2C12 myotubes; C2C12 CM sup, the supernatant from C2C12 myotubes exposed to 20% C26‐conditioned medium (C26‐CM). To prevent nutrient depletion and maintain consistent basal conditions, all supernatants were mixed 1:1 with fresh serum‐free and M‐CSF‐free DMEM before being applied to BMDMs for 24 h.

Flow cytometry analysis revealed a significant increase in CD11c MFI—a marker of M1 polarization—in the C2C12 CM sup group compared to the control and C2C12 DM sup groups (Figure 2B). Conversely, CD206 expression, a marker of M2 polarization, was reduced in the C2C12 DM sup and C2C12 CM sup groups relative to the control. These findings were further validated by qRT‐PCR, which showed a marked elevation in TNF‐α mRNA, a pro‐inflammatory M1‐associated cytokine, in BMDMs treated with C2C12 CM sup (Figure 2C). In contrast, no significant changes were observed in the expression of TGF‐β, an M2‐associated marker, across the groups. Collectively, these results indicate that soluble mediators secreted by cachectic myotubes, rather than cancer‐derived factors alone, primarily drive the polarization of macrophages toward a pro‐inflammatory M1 phenotype.

### 3.4. M1 Macrophages Amplify Inflammatory Cytokine Production in Myocytes

**Figure 3:**
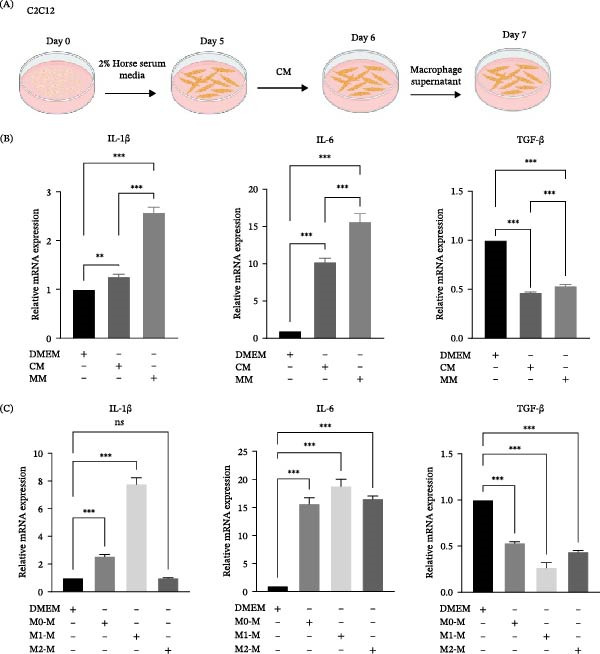
C2C12 myotubes cultured with M1‐polarized macrophage‐conditioned media exhibit elevated pro‐inflammatory cytokine expression. (A) Schematic representation of the experimental design used for the in vitro co‐culture model. (B) mRNA expression levels of pro‐inflammatory cytokines in C2C12 cells 24 h after stimulation with either cancer conditioned media (CM) or macrophage conditioned media (MM). (C) mRNA expression levels of IL‐1β, IL‐6, and TGF‐β in C2C12 cells co‐cultured with conditioned media from macrophages polarized to different phenotypes (M0, M1, or M2). Bars represent the mean values, and the error bars represent the standard error of the mean (SEM) of three independent experiments (*n* = 3). For comparisons between two groups, an unpaired Student’s *t*‐test was used, whereas comparisons involving more than two groups were analyzed using one‐way ANOVA followed by the Dunnett’s post hoc test to assess statistical significance. Statistical significance is indicated as follows:  ^∗^ *p* < 0.05;  ^∗∗^ *p* < 0.01;  ^∗∗∗^ *p* < 0.001; ns = not significant, *p* > 0.05.

An in vitro coculture model was used to evaluate the impact of CC myocyte‐induced M1‐polarized macrophages on inflammatory responses during CC (Figure 3A). C2C12 myoblasts were differentiated into myotubes by culturing in a differentiation medium containing 2% horse serum for 5 days. Exposure of these differentiated myotubes to a tumor cell‐conditioned medium (CM) for 24 h induced atrophy‐related gene expression (Figure S3). To assess cytokine expression, C2C12 cells were subsequently incubated with CM or macrophage medium (MM) for 24 h, and mRNA levels of pro‐inflammatory cytokines, including IL‐1β, IL‐6, and TGF‐β, were quantified (Figure 3B). C2C12 cells cocultured with CM exhibited elevated levels of IL‐1β and IL‐6 compared with those in the DMEM control group. Notably, coculture with MM further enhanced IL‐1β and IL‐6 expression relative to the CM and control groups. In contrast, TGF‐β expression was reduced under both MM and CM conditions compared with that of the DMEM group. These results suggest that macrophage‐derived soluble mediators contribute to the exacerbation of inflammation in cachectic myocytes. To further determine the specific macrophage polarization state responsible for this effect, C2C12 cells were cultured in a MM that had been polarized to the M1 or M2 phenotype (Figure 3C). After polarization, macrophage cultures were washed and maintained in a fresh medium prior to conditioned media collection to eliminate the residual effects of the polarization stimuli. Myocytes cocultured with unpolarized macrophages (M0‐M) showed increased IL‐1β and IL‐6 expressions compared with those cultured in DMEM. Exposure to both M1‐ and M2‐polarized macrophage‐conditioned media resulted in an upregulation of inflammatory markers in myocytes. However, the expression level of IL‐1β and IL‐6 was significantly higher in myocytes treated with M1‐conditioned media compared to those with M2‐conditioned media. These results suggest that while multiple macrophage phenotypes may participate in the inflammatory process, M1‐polarized macrophages serve as the predominant contributors to the exacerbation of pro‐inflammatory cytokine expression in this cachectic myocyte model. Conversely, the TGF‐β expression showed a decreasing trend across all macrophage conditions.

### 3.5. In Vivo Validation of Increased Cytokine Expression in Cachectic Skeletal Muscle

**Figure 4:**
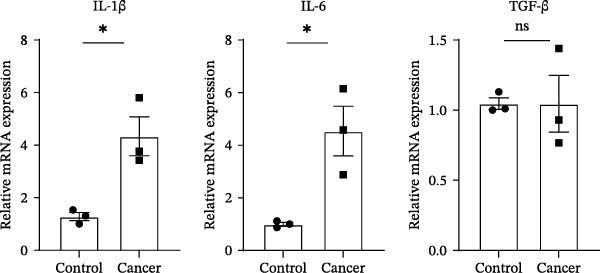
mRNA expression of pro‐ and anti‐inflammatory cytokines in the quadriceps muscles of CC‐affected mice. BALB/c mice were injected with C26 tumor cells to induce CC. After validating the CC model in the full cohort (*n* = 15 per group), relative mRNA expression levels of IL‐1β, IL‐6, and TGF‐β were analyzed by qPCR using randomly selected representative samples (*n* = 3 biological replicates per group). These samples were chosen based on their proximity to the median phenotypic values of the group to ensure accurate representation of the cohort’s biological response. Gene expression was normalized to mean of the control group using the 2^ΔΔCt^ method. All experiments were independently repeated at least three times to ensure reproducibility. Statistical significance between the two groups was determined using an unpaired Student’s *t*‐test or Welch’s *t*‐test, depending on the equality of variances. Statistical significance is indicated as follows:  ^∗^ *p* < 0.05; ns = not significant, *p* > 0.05.

The cytokine expression profile identified in the in vitro model was validated in the skeletal muscles of mice with CC (Figure 4). A significant increase in IL‐1β and IL‐6 mRNA expression was detected in the QC of the cancer group, with levels approximately two‐ to three‐fold higher than those in the control group. In contrast, no significant difference in TGF‐β expression was detected between the two groups, corroborating the in vitro findings.

## 4. Discussion

CC is marked by an ongoing loss of skeletal muscle mass accompanied by systemic inflammatory responses. Evidence indicates that macrophages are central mediators of local inflammatory responses in the muscle tissue. Previous studies have demonstrated that immune cell infiltration, particularly by macrophages, contributes to muscle degeneration and atrophy in various pathological conditions, including cancer [34–36]. In this study, we examined the inflammatory profiles of macrophages infiltrating skeletal muscle and explored their paracrine interactions with myocytes in a mouse model of CC. The conditioned medium derived from C2C12 myotubes exposed to cancer‐conditioned media (CM) was sufficient to induce the polarization of BMDMs toward an M1‐like phenotype.

Although most existing literature focuses on the influence of tumor‐derived factors or tumor‐conditioned macrophages on myocytes, our study highlights a distinct “reverse” feedback loop, wherein atrophying myocytes themselves serve as a key source of inflammatory cues. This bidirectional cross talk suggests that skeletal muscle is not merely a passive target of systemic inflammation but an active participant in modulating the local immune microenvironment during CC progression. These findings suggest that soluble mediators secreted by stressed or atrophying myotubes can influence macrophage polarization in a contact‐independent manner. Although IL‐1β emerged as a potential effector in this model, other cytokines or damage‐associated molecular patterns (DAMPs) also likely contribute to macrophage activation in this context [37–40]. M1‐polarized macrophages are well recognized for their pro‐inflammatory profile, including the secretion of cytokines, such as TNF‐α, which promote muscle atrophy through the upregulation of proteolytic pathways (e.g., MuRF1 and Atrogen‐1) and suppression of myogenetic differentiation via MyoD inhibition [41, 42]. Although macrophage‐specific cytokine secretion was not directly measured in this study, owing to limited yields of isolated cells, cytokine expression was analyzed in whole muscle tissues, and in vitro coculture models were used to probe macrophage–myocyte interactions. Future studies should aim to isolate and characterize these muscle‐infiltrating macrophage populations directly to confirm their secretory profiles.

Collectively, these experimental strategies support the hypothesis that M1 macrophages play a contributory role in amplifying local inflammation and muscle degeneration in CC. Notably, the fold changes in pro‐inflammatory gene expression, such as TNF‐*α*, observed in our in vitro models were relatively modest compared to the robust responses typically induced by potent stimuli like LPS. However, unlike the acute and intense inflammatory burst seen in sepsis models, CC is characterized by chronic, low‐grade systemic inflammation [31, 43]. In this chronic pathological context, even a sustained low‐level elevation of pro‐inflammatory cytokines can be sufficient to activate muscle proteolytic pathways and impair myogenic differentiation over time.

Thus, our findings represent a pathophysiological relevant state of macrophage activation that reflects the actual microenvironment of cachectic muscle. This is particularly true for the in vivo observations shown in Figure 4. In the complex microenvironment of cachectic muscle, a two‐ to three‐fold chronic elevation of cytokines such as IL‐1β and IL‐6 is well documented to be sufficient for the persistent activation of the JAK/STAT and NF‐κB pathways, leading to muscle protein degradation [44–46]. Therefore, the magnitude of these changes should be interpreted not as insufficient compared with acute inflammatory models but rather as a consistent and cumulative signal that underlies the progressive nature of CC.

Given the strong association between skeletal muscle loss and poor clinical outcomes in patients with cancer [47, 48], a deeper understanding of the macrophage‐mediated inflammatory process is critical for developing targeted interventions. Future studies should profile cytokine expression at the single‐cell level, particularly within macrophage subsets infiltrating the cachectic muscle tissue.

The implications of these findings may extend beyond CC. Similar immune‐driven mechanisms have been implicated in other muscle‐wasting disorders, such as Duchenne muscular dystrophy [49]. CD163‐positive macrophages have also been identified in the skeletal muscles of patients with pancreatic ductal adenocarcinoma, suggesting that immune‐mediated muscle wasting may be a shared feature across different cancer types [26]. However, heterogeneity in tumor biology, treatment history, and host age must be considered when interpreting immune phenotypes in human studies.

Furthermore, while our in vitro results provide a mechanistic basis for cross talk, they may not fully capture the complex systemic environment of CC. Future studies involving the depletion of specific macrophage subsets in tumor‐bearing mice will be essential to validate these findings in vivo. This study has several limitations. First, we utilized CD11c and CD206 as primary markers to define M1‐ and M2‐like macrophage populations, respectively. Although these are widely accepted markers for assessing the predominant inflammatory shift in various muscle‐wasting models [50], acknowledging that in vivo macrophage phenotypes exist as a continuous spectrum of activation rather than a strict binary classification is important. Skeletal muscle macrophages in the cachectic niche are highly plastic and may exhibit hybrid functional states that are not fully captured by single‐marker profiling. Future studies employing a broader panel of markers (e.g., iNOS or CD80 for M1 and Arg1 or CD163 for M2) are required to fully elucidate the complex landscape of these infiltrating immune cells. Additionally, while we focused on mRNA expression as a surrogate for the inflammatory state, we recognize that transcriptional changes do not always directly correlate with protein secretion levels. Although sample constraints precluded extensive cytokine protein quantification via ELISA, our flow cytometry data confirm a significant increase in the infiltration of pro‐inflammatory myeloid cells, which are known to be the major producers of these cytokines. Future investigations incorporating secretome analysis via multiplex assays will be essential to precisely define the cytokine flux within the cachectic muscle niche. Furthermore, CD206 is also expressed by certain tissue‐resident macrophage subsets, which complicates the distinction between resident and alternatively activated (M2) macrophages [51]. Although young mice (5–8 weeks old) were used to minimize biological variability, age‐dependent differences in immune responses warrant further investigation [52, 53]. Cachexia severity is also influenced by strain‐dependent factors, tumor burden, and the timing of endpoint analyses. In this study, the tumor mass was included in total body weight measurements, in line with established practices [54]; however, this approach should be interpreted with caution when assessing cachexia progression.

## 5. Conclusions

This study suggests that inflammation‐associated macrophages, particularly those polarized toward the M1 phenotype, may play a role in amplifying skeletal muscle inflammation and cytokine production during CC. These findings highlight the potential importance of macrophage–myocyte cross talk in the cachectic muscle microenvironment. Notably, our in vitro observations do not account for the systemic complexities of CC. Additional studies using ex vivo skeletal muscle macrophages and in vivo models are essential to further validate these mechanisms. Ultimately, elucidating this dynamic signaling loop will be essential for developing targeted therapeutic strategies aimed at preserving muscle mass in CC.

## Funding

This study was supported by grants from the National Research Foundation of Korea (NRF), which is funded by the Korean government (MSIT) (Grants RS‐2023‐00220894 and 2023R1A2C1006127).

## Ethics Statement

All procedures involving animals were approved by the Institutional Animal Care and Use Committee of Korea University (Approval Number KOREA‐2023‐0015) and were carried out in compliance with established protocols and regulations.

## Conflicts of Interest

The authors declare no conflicts of interest.

## Supporting Information

Additional supporting information can be found online in the Supporting Information section.

## Supporting information


**Supporting Information** Figure S1: Characterization of C26 adenocarcinoma–induced cancer cachexia (CC) in mice. Figure S2: Flow cytometric analysis of lymphocyte populations in cancer cachexia (CC)–affected mouse skeletal muscle. Figure S3: Muscle atrophy phenotype of C2C12 myotubes following incubation with cancer cell supernatants.

## Data Availability

The data that support this study’s findings are available from the corresponding author upon request.
