# Supplementary material for: Macrophage–Myocyte Cross Talk Induces M1 Polarization and Inflammatory Cytokine Production During Cancer Cachexia
Source: Mediators Inflamm. 2026 Jul 14;2026:6461737. doi: 10.1155/mi/6461737 (PMC13369589; doi:10.1155/mi/6461737)
Supplement: Supplementary file 1 — Supporting Information Figure S1: Characterization of C26 adenocarcinoma–induced cancer cachexia (CC) in mice. Figure S2: Flow cytometric analysis of lymphocyte populations in cancer cachexia (CC)–affected mouse skeletal muscle. Figure S3: Muscle atrophy phenotype of C2C12 myotubes following incubation with cancer cell supernatants. [file MI-2026-6461737-s001.docx]

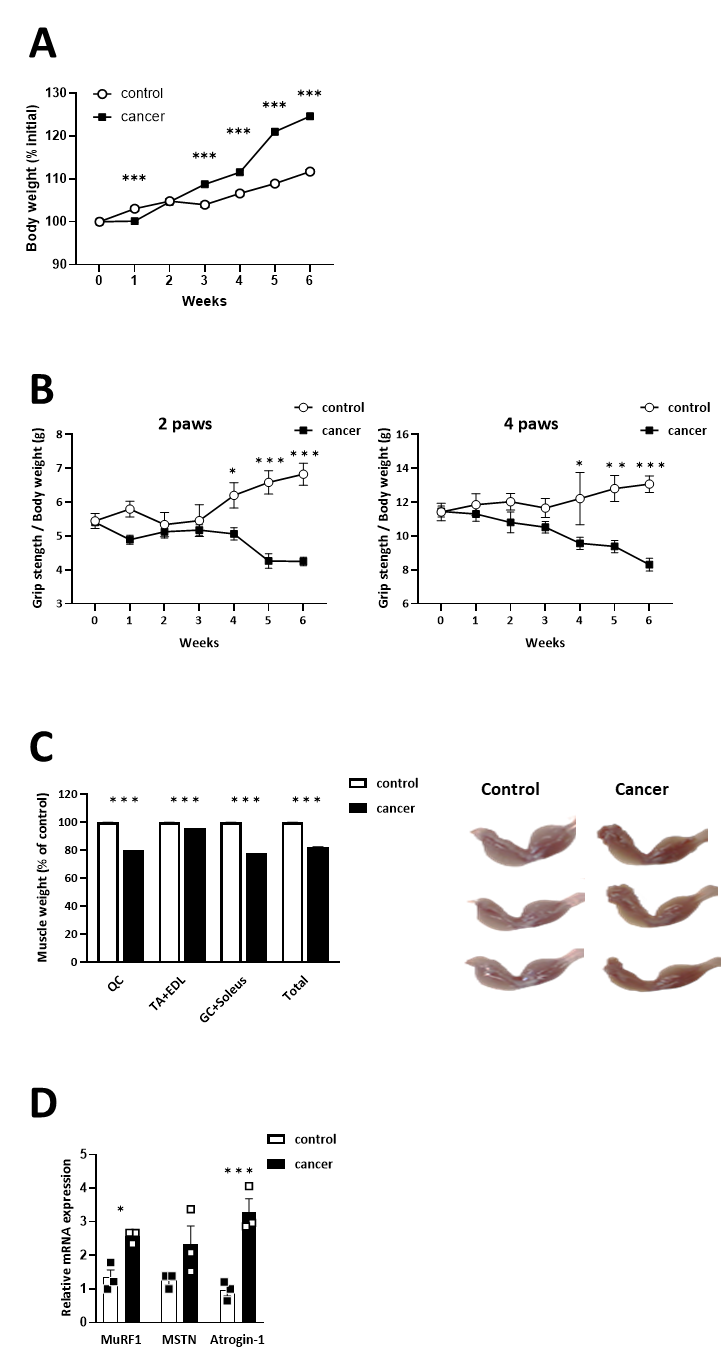
Supplementary material

Figure S1. Characterization of C26 adenocarcinoma-induced cancer cachexia (CC) in mice. (A–D) BALB/c mice (*n* = 15 per group) were subcutaneously injected with C26 tumor cells to induce CC. Skeletal muscles were harvested 6 weeks after injection. (A) Changes in total body weight, including tumor mass, were recorded from baseline. (B) Grip strength measurements (2-paw: forelimb; 4-paw: all limbs) were obtained weekly. (C) Muscle weights and representative images of the hindlimb were collected from tumor-bearing and control mice. (D) Relative mRNA expression levels of muscle atrophy-related genes, muscle RING-finger protein-1 (MuRF1), atrogin-1, and MSTN in the quadriceps (QC) muscles of control and tumor-bearing mice (*n* = 5 per group). Gene expression levels were normalized to GAPDH expression. qPCR was performed in triplicate, and the average Ct values were used for analysis. Data are presented as the mean ± standard error of the mean (SEM). Statistical significance was determined using an unpaired Student’s *t*-test for comparisons between two groups. Significance is indicated as follows: **p* < 0.05; ***p* < 0.01; and ****p* < 0.001.


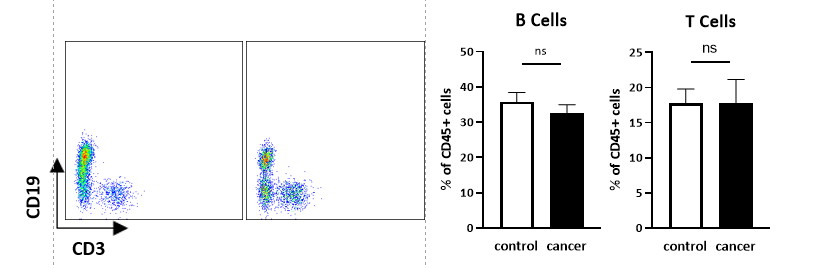
Figure. S2. Flow cytometric analysis of lymphocyte populations in mouse skeletal muscle with cancer cachexia (CC). Representative flow cytometry plots and quantification of T cells (CD45^+^CD3^+^) and B cells (CD45^+^CD19^+^) in the skeletal muscle of control and CC-affected mice. Statistical significance was determined using an unpaired Student’s *t-*test for comparisons between two groups and indicated as ns = not significant (*p* > 0.05).


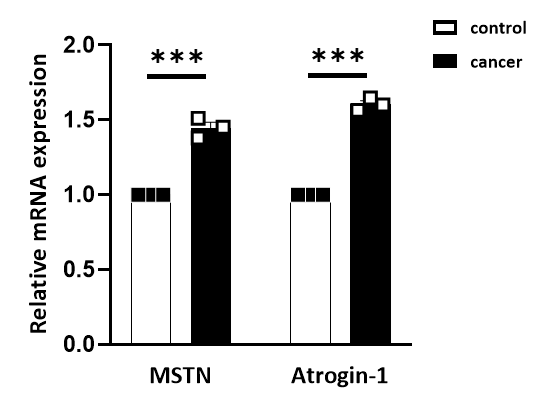


Figure. S3. Muscle atrophy phenotype of C2C12 myotubes after incubation with cancer cell supernatants. C2C12 cells were differentiated into myotubes and incubated with cancer cell supernatants for 24 h. The mRNA expression levels of the muscle atrophy-related genes myostatin (MSTN) and atrogin-1 were quantified by qPCR. The bars represent the mean values, and the error bars represent the standard error of the mean (SEM). Statistical significance was determined using an unpaired Student’s *t-*test for comparisons between two groups (*** *p* < 0.001).
